# *Periplaneta americana* Arginine Kinase as a Major Cockroach Allergen among Thai Patients with Major Cockroach Allergies

**DOI:** 10.1289/ehp.8650

**Published:** 2006-01-26

**Authors:** Nitat Sookrung, Wanpen Chaicumpa, Anchalee Tungtrongchitr, Pakit Vichyanond, Chaweewan Bunnag, Pongrama Ramasoota, Pongsri Tongtawe, Yuwaporn Sakolvaree, Pramuan Tapchaisri

**Affiliations:** 1 Graduate Studies, Faculty of Allied Health Sciences, Thammasat University, Rangsit Center, Pathum-thani, Thailand; 2 Department of Parasitology, Faculty of Medicine, Siriraj Hospital, Mahidol University, Bangkok, Thailand; 3 Department of Pediatrics, Faculty of Medicine, Siriraj Hospital, Mahidol University, Bangkok, Thailand; 4 Department of Otorhinolaryngology, Faculty of Medicine, Siriraj Hospital, Mahidol University, Bangkok, Thailand; 5 Department of Social Medicine and Environment, Faculty of Tropical Medicine, Mahidol University, Bangkok, Thailand

**Keywords:** allergen, arginine kinase, IgE, immunoblotting, *P. americana*, peptide phage display, proteomics, two-dimensional gel electrophoresis

## Abstract

*Periplaneta americana* is the predominant cockroach (CR) species and a major source of indoor allergens in Thailand. Nevertheless, data on the nature and molecular characteristics of its allergenic components are rare. We conducted this study to identify and characterize the *P. americana* allergenic protein. A random heptapeptide phage display library and monoclonal antibody (MAb) specific to a the *P. americana* component previously shown to be an allergenic molecule were used to identify the MAb-bound mimotope and its phylogenic distribution. Two-dimensional gel electrophoresis, liquid chromatography, mass spectrometry, peptide mass fingerprinting, and BLAST search were used to identify the *P. americana* protein containing the MAb-specific epitope. We studied the allergenicity of the native protein using sera of CR-allergic Thai patients in immunoassays. The mimotope peptide that bound to the MAb specific to *P. americana* was LTPCRNK. The peptide has an 83–100% identity with proteins of *Anopheles gambiae*, notch homolog scalloped wings of *Lucilia cuprina*, delta protein of *Apis mellifera*; neu5Ac synthase and tyrosine phosphatase of *Drosophila melanogaster*, and a putative protein of *Drosophila pseudoobscura*. This finding implies that the mimotope-containing molecule of *P. americana* is a pan-insect protein. The MAb-bound protein of *P. americana* was shown to be arginine kinase that reacted to IgE in the sera of all of the CR-allergic Thai patients by immunoblotting, implying its high allergenicity. In conclusion, our results revealed that *P. americana* arginine kinase is a pan-insect protein and a major CR allergen for CR-allergic Thai patients.

Cockroach (CR) allergy has been recognized as important IgE-mediated type 1 hypersensitivity since 1964 (Bernton and Brown 1964). Prolonged exposure and sensitization to CR allergen, which is one of the major indoor allergens in Thailand, give rise to asthmatic attacks that require emergency hospital visits, especially in young children (Chapman et al. 1996; Tungtrongchitr et al. 2004). Because CRs are ubiquitous, CR allergy is a worldwide public health problem, especially in cities with dense housing and a crowded population where the living conditions favor CR infestation (Chapman et al. 1996; Sarpong et al. 1996). It is now accepted that there is a relationship between CR exposure, CR allergen sensitization, and asthma (Eggleston et al. 1999; Sporik et al. 1999). Avoiding exposure to the causative allergens is the most effective measure to reduce asthmatic morbidity (Call et al. 1992; Gelber et al. 1993). Thus, the quantitative determination of indoor allergens including CR allergens is the most important measure for disease intervention.

Most reagents currently available for quantitation of CR allergens are aimed at the German CR allergens (Platts-Mills et al. 1997). In 2003, two murine hybridomas, clones 3C2 and 38G6, secreting specific monoclonal antibodies (MAb) to allergens derived from the American cockroach, *Periplaneta americana*, were produced in our laboratory (Sookrung et al. 2003). Affinity-column–purified antigens of the crude American CR extract reactive to the MAb secreted by the two hybridoma clones gave positive Prausnitz-Kustner (P-K) tests. For the P-K test, pooled sera of CR-allergic Thai patients was injected into shaved skin of a guinea pig at multiple sites; after 24 hr the same sites were individually injected with either the affinity-purified CR antigens or phosphate-buffered saline (PBS), which was used as a negative control. Wheal and flare were observed at the sites injected with the CR antigens 15 min after the injection but not at the sites injected with the negative control, implying the allergenic property of the two affinity-purified CR antigens (Sookrung et al. 2003). The antigen specific to MAb3C2 proved to be a gene product of Per a 1 variant (Per a 1.0105; GenBank accession no. AY 259514; http://www.ncbi.nih.gov/Genbank/) (Diraphat et al. 2003). The deduced amino acid sequence of the gene encoding Per a 1.0105 (372-bp open-reading frame), which is a 124-amino-acid protein with a molecular mass of 13.8 kDa and an isoelectric point (pI) of 4.74, revealed that the molecule contained a mitochondrial energy transfer protein signature, that is, phosphorylation sites for cAMP- and cGMP-dependent protein kinase C and casein kinase II. Hydrophobic and hydrophilic characteristics of the deduced polypeptide indicated that it was a transmembrane protein orthologous to a putative protein in the midgut of *Aedes aegypti* (*G12*; GenBank accession no. AY050565; http://www.ncbi.nih.gov/Genbank/) (Diraphat et al. 2003). The MAb of clone 3C2 specific to Per a 1.0105 was used in a sandwich ELISA as a detection reagent for *P. americana* allergen in the homes of CR-allergic Thai patients (Tungtrongchitr et al. 2004).

In this study we used a random heptapeptide phage display library to identify the mimotope of the MAb38G6. Moreover, *P. americana* protein containing the MAb38G6 epitope was characterized using two-dimensional gel electrophoresis, two-dimensional immunoblotting, liquid chromatography–mass spectrometry (LC-MS), MS–peptide mass fingerprinting (PMF), and BLAST (Basic Local Alignment Search Tool; NCBI 2005) search. The molecule was found to be *P. americana* arginine kinase, which reacted to IgE in the sera of all CR-allergic Thai patients. Details of our experiments form the basis of this report.

## Materials and Methods

### Human subjects and sera

We collected sera individually from 25 Thai patients with CR allergy who gave a positive skin prick test to crude *P. americana* extract. They were patients of the Department of Otorhinolaryngology, Faculty of Medicine, Siriraj Hospital, Mahidol University in Bangkok, Thailand. We tested serum samples of some patients for specific IgE antibodies against German CR extract using the CAP system (Amersham Biosciences, Uppsala, Sweden). Cutoff point between CAP positive and negative was 0.35 IU. Sera of five individuals with a negative skin prick test to crude *P. americana* extract served as controls. The patients and the controls gave their informed consent. Listed in Table 1 are the characteristics of the 25 allergic patients and the five negative controls.

### *MAb specific to the* P. americana CR *allergen*

MAb specific to *P. americana* antigen (allergen) secreted by hybridoma clone 38G6 (Sookrung et al. 2003) grown in a serum-free medium (Gibco, Invitrogen Corp., Grand Island, NY, USA) were used.

### Preparation of *P. americana* crude extract

Adult *P. americana* caught from houses in Bangkok were entomologically identified and kept frozen at −70°C until used. The frozen *P. americana* were ground to fine pieces in liquid nitrogen using mortar and pestle. PBS (0.15 M), pH 7.4, containing protease inhibitors (Roche Diagnostics GmbH, Mannheim, Germany) was added; the preparation was sonicated (model VC750; Vibra Cell Sonics and Materials Inc., Newtown, CT, USA) at a 30% amplitude, 2-sec pulse on, 2.5-sec pulse off, for a total of 5 min and then centrifuged at 12,000 ×*g*, at 4°C, for 20 min. The supernatant (CR extract) was collected, and the protein content was quantitated using Bradford reagents (Bio-Rad, Hercules, CA, USA) (Bradford 1976).

### T7 heptapeptide phage peptide library

A random heptapeptide phage display library was constructed by G. Froman (Department of Medical Microbiology, Uppsala University, Sweden) using the T7 select-415 kit from Novagen (Madison, WI, USA). The library construction was started by synthesizing a random heptapeptide DNA. The DNA sequences were derived from degenerate oligonucleotides, which were synthesized chemically by adding mixtures of nucleotides to growing nucleotide chains. The oligonucleotide synthesis was designed to yield seven-residue-long random amino acid sequences flanked by cysteine residues. To limit the occurrence of in-frame stop codons, the third position of every nucleotide triplet after the first cysteine codon was synthesized from a mixture of G and T nucleotides. A mixture of 32 nucleotide triplets was formed, including codons for all 20 natural amino acids, and one stop codon. Each synthesized oligonucleotide construct was ligated to the T7 vector arm. The target peptide was expressed as a fusion partner to the C-terminus of the major capsid protein and was displayed on the virion surface, where it was accessible for interaction with other proteins or ligands. Each displayed peptide was situated between cysteine residues, and therefore, formation of a disulfide bridge joined the ends of the heptapeptide. The fusion peptide was present in 415 copies on each phage particle. The library was designated T7S. It had an original size of 3.3 × 10^7^ pfu, but it was amplified to a titer of 2.6 × 10^10^ pfu/mL before use (Houshmand et al. 1999).

### Biopanning for the determination of the MAb38G6 mimotope

MAb38G6 was diluted in PBS to 10 μg/mL; 100-μL aliquots were distributed to the wells of a 96-well microtiter plate (Costar, Corning, NY, USA) and incubated at 37°C overnight. The MAb-coated wells were washed with PBS containing 0.05% Tween-20 (PBST), blocked by adding 200 μL PBS containing bovine serum albumin (BSA) (50 mg/mL) to each well, and incubated at 4°C for 18 hr. Excess blocking reagent was removed by washing with PBST; the T7S-peptide phage library was added to the MAb-coated wells and incubated at 25°C under agitation for 30 min. Unbound phages were washed off; bound phages were released by incubating each well with 100 μL of 1% SDS. The eluted phages were used to infect *Escherichia coli* BL21 cells to produce a peptide-phage sublibrary for the next biopanning round. Three more biopanning rounds were done to increase the phage binding affinity to the MAb. Finally, the phages in the forth sublibrary were cloned by plaque isolation, and 10 single plaques of T7 phages were randomly picked by cutting out the gel plugs. DNA was extracted from individual gel plugs containing the selected phages and used as templates for DNA amplification by polymerase chain reaction (PCR).

### PCR and DNA sequencing

DNA of the selected phage clones were amplified according to the instructions of the manufacturer (Novagen, Madison, WI, USA) using the T7 select up (5′-AGCTGTCGTATTCCAGT CA-3′) and down (5′-ACCCCTCAAGACCC GTTTA-3′) nucleotides as primers. The PCR product was purified using the QIA quick PCR purification kit (Qiagen, Valencia, CA, USA). The product, together with the T7 selected up primers, was then subjected to an automated DNA sequencing procedure.

### Two-dimensional gel electrophoresis

American CR extract was cleaned with the 2D-Clean-up kit (Amersham Biosciences, San Francisco, CA, USA) to eliminate detergents, salts, lipids, phenolics, and nucleic acids. After cleaning, the preparation was rehydrated in a buffer containing immobilized pH gradient (IPG) buffer (8 M urea, 2% 3-[(3-cholamido-propyl)-dimethylammonio]-1-propane sulfonate, 0.5% IPG buffer 3–10 nonlinear, 0.002% bromophenol blue). The protein content was determined using the 2D-Quant kit (Amersham Biosciences) before subjecting it to the first-dimensional electrophoresis. The preparation (60 μg protein) was added to DeStreak Rehydration Solution containing IPG (0.5% IPG buffer 3–10 nonlinear; Amersham Biosciences, Uppsala, Sweden). The preparation was loaded into the IPG strip holder, and care was taken not to produce any bubbles. The IPG strip, stored at −20°C, was placed right side down into the strip holder containing the sample, and dry strip cover fluid (1 mL) was added. The strip holder was placed into the Ettan IPG Phor Electrofocusing System (Amersham Biosciences, Uppsala, Sweden), and the IPG strip was allowed to rehydrate at 20°C for 12 hr. Electrophoresis was performed initially at 0.2 kV/hr for 30 min, followed by 0.3-kV/hr gradient for 30 min, 4.0 kV/hr for 80 min, and step and hold for 15 min. For the two-dimensional gel electrophoresis, the electrofocused IPG strip was equilibrated in 10 mL SDS equilibration buffer [50 mM Tris-HCl (pH 8.8), 6 M urea, 30% glycerol, 2% SDS, 0.002% bromophenol blue] containing 100 mg dithiothreitol for 15 min. Subsequently, the strip was placed in 10 mL of the equilibration buffer containing 250 mg iodoacetamide for 15 min. It was washed with electrode buffer and overlaid onto a 12% polyacrylamide gel casted in Mini-PROTEAN 3 Cell (Bio-Rad). SDS-PAGE was performed at 10 mA/gel during the first 15 min and at 20 mA/gel until the tracking dye reached the lower edge of the gel. After SDS-PAGE, the gel was either stained by Coomassie brilliant blue (CBB) dye for the CR proteomics, or the separated components in the gel were electrotransblotted onto a polyvinylidene difluoride (PVDF) membrane for further identification by two-dimensional immunoblotting with MAb38G6.

### Two-dimensional immunoblotting

The PVDF membrane blotted with the two-dimensional-gel–separated CR components was placed in a blocking solution [3% BSA in Tris-buffered saline, pH 7.4 (TBS)] at 25°C for 1 hr. After washing with a wash buffer [TBS containing 0.05 Tween-20 (TBS-T)], the blot was incubated with MAb38G6 at 25°C for 2 hr. After the excess MAb was removed and the membrane was washed thoroughly with the wash buffer, it was placed in a rabbit anti-mouse immunoglobulin–biotin conjugate (DakoCytomation, Glostrup, Denmark; diluted 1:1,000 in TBS-T) at 25°C for 1 hr, washed with TBS-T, and incubated with streptavidin-alkaline phosphatase (DakoCytomation), diluted 1:1,000, in a diluent (0.2% BSA, 0.2% gelatin in TBS) at 25°C for 30 min. After the membrane was washed with the wash buffer and placed in 0.15 M Tris-HCl (pH 9.6) for 10 min, it was then incubated with bromo-4-chloro-3-indoxyl-phosphate/nitroblue tetrazolium (BCIP/NBT) substrate (KPL, Gaithersburg, MD, USA) in the dark for 10 min. The enzyme–substrate reaction was stopped by rinsing the membrane with deionized distilled water.

### Liquid chromatography-mass spectrometry

Protein spots corresponding to those reactive to MAb38G6 in the two-dimensional immunoblot were cut from the two-dimensional CBB-stained gel. They were subjected to in-gel reduction, alkylation, and trypsin digestion. For LC-MS analysis, a nano-LC system (Finnigen) was coupled to a Finnigen LTQ (Thermo Electron Corp., Waltham, MA, USA). Peptide separation was done using a C_18_ reversed-phase column (BioBasic-18; column dimensions, 100 × 0.18 mm; 5-μm particle size) and a gradient of 0–60% B in A (A = 0.1% formic acid in water; B = 0.1% formic acid in acetonitrile) for 22 min, at a constant flow rate of 200 μL/min. Each digest was analyzed in LC-MS mode for identification. Fragmentation of the peptides was performed in data-dependent mode, and mass spectra were acquired in continuum mode.

### Mass spectrometry and peptide mass finger-printing

A protein spot, corresponding to its counterpart reactive to MAb38G6 in the two-dimensional immunoblot, was cut from the two-dimensional CBB-stained gel and destained with 50 mM ammonium bicarbonate containing 50% methanol. This process was repeated twice to completely destain, to wash out traces of salts, and to change the pH. After destaining, the gel plug was air dried at 25°C until the plug appeared white and powdery. Trypsin (20–40 ng/μL in 20 mM ammonium bicarbonate) was added in a small volume (< 5 μL) to get as much as possible inside the gel plug during the rehydration. Trypsin digestion was performed at 25°C for 1.5 hr. The digested sample was transferred to the extraction plate by adding 25 μL 50% acetonitrile, 0.5% TFA solution (TFA/anisole/ethylmethylsulfide/ethanedithiol in the proportion 93:3:3:1, respectively, by volume). The solution was run up and down in the pipette tip before transferring it to the new tube. This procedure was repeated to maximize the extraction process. The sample was spotted onto the MALDI (matrix-assisted laser desorption/ionization) target by mixing the extracted and concentrated sample in a 1:1 ratio (vol/vol) with a 3-mg/mL solution of recrystallized α-cyano matrix. The sample was allowed to air dry before PMF was performed on the sample both with and without internal calibration with Ang III (*m*/*z* 897.523) and adrenocorticotropic hormone (*m*/*z* 2465.19). All results were obtained on the Ettan MALDI-TOF (time of flight) Pro (Oracle 9i softtware; Amersham Biosciences, Uppsala, Sweden) using methods preprogrammed into the Ettan control window. The resultant spectra were the sum of 200 laser shots.

### MAb affinity-purified CR antigen

Protein of the *P. americana* that reacted to MAb38G6 that had previously been shown to be an allergen was prepared from the CR crude extract using MAb affinity column chromatography as previously described (Sookrung et al. 2003). The affinity-purified allergen was used in a Western blot analysis and in sandwich ELISA for detecting IgE in the sera of patients and controls.

### SDS-PAGE and Western blot analysis

The affinity-purified *P. americana* protein (50 μg) was separated in a 12% SDS-PAGE in a Mini-PROTEAN 3 Cell (Bio-Rad) and electroblotted onto a PVDF membrane (Amersham Biosciences, Bucks, UK) that was cut into vertical strips. The strips were blocked with 3% BSA in TBS at 25°C for 1 hr and then incubated individually with the sera of CR-allergic patients or non-CR-allergic controls (diluted 1:10 in diluent, i.e., 0.2% BSA, 0.2% gelatin in TBS) at 4°C overnight. After rinsing with TBS-T, the strips were placed in a solution of mouse anti-human IgE–biotin conjugate (Zymed Laboratory, San Francisco, CA, USA) diluted 1:1,000 and incubated at 25°C for 3 hr, washed with TBS-T, incubated with streptavidin–alkaline phosphatase conjugate (DakoCytomation) diluted 1:1,000 in diluent at 25°C for 30 min, washed, and then placed in 0.15 M Tris-HCl (pH 9.6) for 10 min. The strips were finally incubated with BCIP/NBT substrate (KPL) in the dark for 10 min. The enzyme–substrate reaction was stopped by rinsing all strips with deionized distilled water.

### ELISA

A sandwich ELISA was used for determining the allergenicity of the *P. americana* protein containing the MAb38G6-specific epitope. One hundred microliters of MAb38G6 (40 μg/mL in carbonate-bicarbonate buffer, pH 9.6) was added to each well of the ELISA plate (Costar). The plate was incubated at 37°C until it was dry. All coated wells were washed with PBST; 200 μL of a blocking solution (1% BSA in PBS, pH 7.4) was added individually and the plate was further incubated at 37°C for 1 hr. After washing with PBST, 100 μL of the affinity-purified *P. americana* antigen (5 μg/mL) was added to each well, which were then incubated at 37°C for 30 min and washed as above. Then, 100 μL of the individual patient serum was added to the wells. A well with PBS added served as a blank. The ELISA plate was incubated at 4°C overnight and washed thoroughly with PBST. One hundred microliters of mouse anti-human IgE–biotin conjugate (Zymed Laboratory; diluted 1:1,000 in diluent) was added to each well. The plate was incubated at 37°C for 3 hr and washed with PBST, and 100 μL of streptavidin–horseradish peroxidase (DakoCytomation) diluted 1:1,000 in the diluent was added to each well. After further incubating at 37°C for 30 min, the plate was washed as above, and freshly prepared substrate [*O*-phenylenediamine (Zymed Laboratory) in a citrate-phosphate buffer, pH 5.0, containing 0.03% hydrogen peroxide] was added to each well (100 μL/well). The plate was kept in the dark at 25°C for 30 min, after which the reaction was stopped by adding 50 μL of 4 N H_2_SO_4_. The optical density (OD) of the content of each well was measured at 492 nm with an ELISA reader (Multiscan EX, Labsystems, Helsinki, Finland) against the blank.

### Statistical analysis

Mann-Whitney rank sum test was used for comparing ELISA OD of arginine kinase specific IgE in sera of patients with CR allergy and normal controls. *p*-Value < 0.01 is statistically significant.

## Results

### Mimotope of MAb38G6 identified by T7 heptapeptide phage display library

DNA extracted from the 10 T7 phage clones derived from the fourth phage sublibrary with high binding affinity to the MAb38G6 were PCR amplified, and DNA of the individual amplicons was sequenced. All clones were found to have the same nucleotide sequence of CTC ACT CCA TGC CGT AAT AAG; thus, the peptide sequence was LTPCRNK. The LTP were amino acids of the T7 capsid peptide (Houshmand et al. 1999).

Polypeptides in the database matching to the mimotope LTPCRNK are shown in Table 2. An 83–100% identity was found between the LTPCRNK mimotope and proteins of *Anopheles gambiae*, notch homolog scalloped wings of sheep blow flies (*Lucilia cuprina*), neu5Ac synthase and protein tyrosine phosphatase of *Drosophila melanogaster*, putative protein of *Drosophila pseudoobscura*, and the delta protein of the honey bee (*Apis mellifera*).

### Results of two-dimensional immuno-blotting

**Figure 1:**
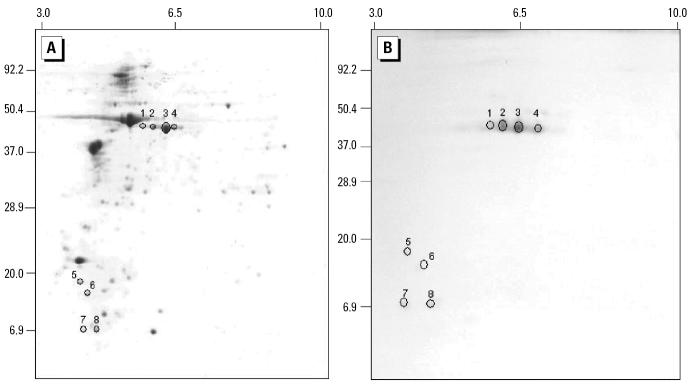
Two-dimensional gel electrophoresis of crude extract of *P. americana* stained by CBB (*A*) and the blot probed with MAb38G6 (*B*). Circles and the indicated numbers in *A* are protein spots that were subjected to LC-MS. Circles in *B* are proteins that reacted to MAb38G6.

Figure 1 shows the proteome of *P. americana* extract after two-dimensional gel electrophoresis stained by CBB dye (Figure 1A) and eight protein spots reactive with MAb38G6 in the two-dimensional immunoblotting (Figure 1B).

### Results of protein identification by LC-MS and MS-PMF

**Figure 2:**
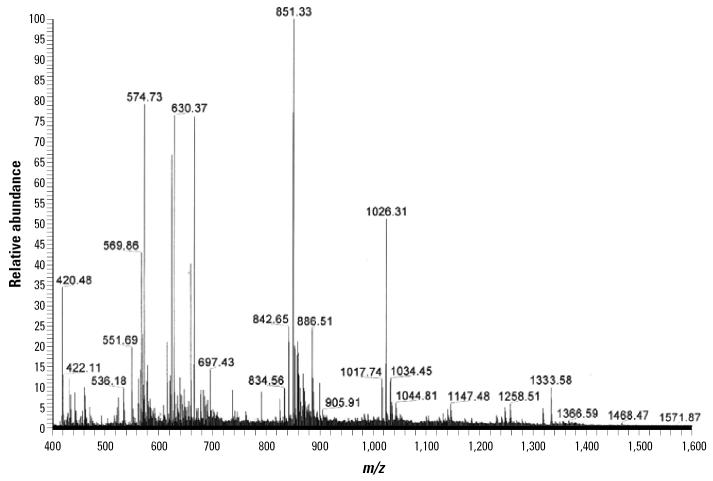
Mass spectra of protein spot 2 (see Figure 1) obtained from LC/MS analysis. The mass spectrometer operated in full-scan mode; the total ion chromatogram was collected over a range of *m*/*z* 400–1,600.

Proteins in gel plugs, corresponding to spots 1–8 in Figure 1B, cut from the two-dimensional gel stained by CBB were subjected to LC-MS. Their amino acid sequences were aligned with those of the proteins deposited in the database. Results showed that the proteins in all of the eight spots were homologous to the protein arginine kinase, which had a pI of 6.3 and a molecular mass of 40.57 kDa. Figure 2 shows the mass spectrum of the protein of spot 2 obtained from LC-MS.

**Figure 3:**
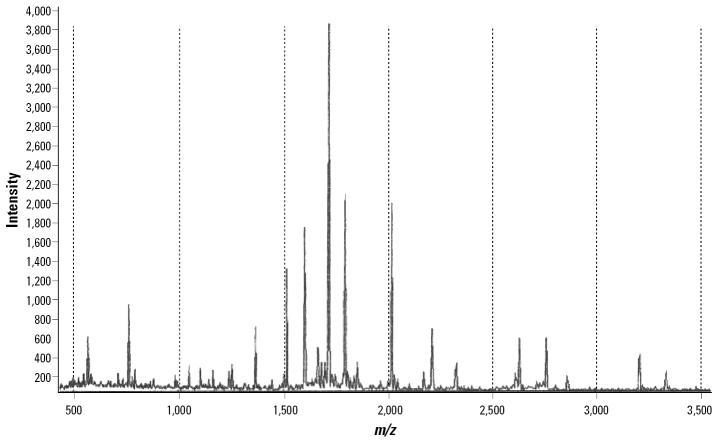
MALDI-TOF MS profile of a tryptic digest of proteins in the gel plug of spot 2. The protein was digested by trypsin; the peptides were analyzed by MALDI-TOF Pro, and the prominent mass peaks were chosen for database searches.

The protein in the gel plug at spot 2 (the most intense protein spot) was also subjected to MS-PMF. The MS profile of the peptide from spot 2 showed multiple peaks ranging from 500 to 3,500 Da (Figure 3). The prominent peaks were selected for comparison with the established databases, and the protein with the highest correlation with this spot was found to be KARG_PACMR arginine kinase. Thus, the results of MS-PMF confirmed those of the LC-MS.

### *Allergenicity of* P. americana *arginine kinase among the CR-allergic Thai patients.*

**Figure 4:**
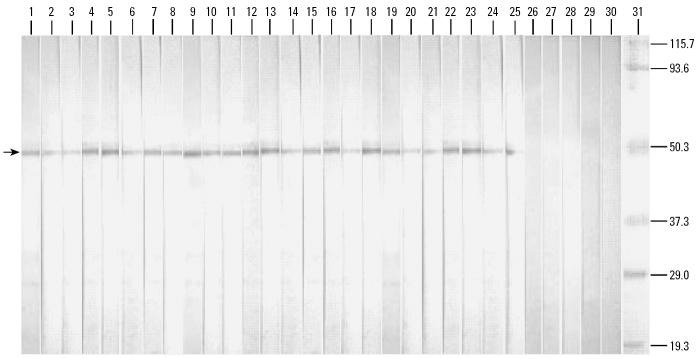
Results of IgE immunoblotting of sera of 25 CR-allergic Thai patients and five controls against *P. americana* arginine kinase prepared from an MAb38G6-affinity column. Lanes 1–25, arginine kinase blots probed with sera of 25 CR-allergic patients; lanes 26–30, arginine kinase blots probed with sera of the five normal controls; lane M, molecular mass marker. Numbers at the right are molecular masses (in kDa). Arrowhead indicates reactive band of arginine kinase and specific IgE in patients’ sera.

SDS-PAGE and Western blot analysis showed that all (100%) of the 25 serum samples of the CR-allergic Thai patients had specific IgE that bound to the MAb38G6 affinity-purified *P. americana* protein, that is, arginine kinase, in their sera (lanes 1–25, Figure 4). None of the negative control serum samples was positive by the test (lanes 26–30, Figure 4).

### *Allergenicity of* P. americana *arginine kinase determined by sandwich ELISA.*

**Figure 5:**
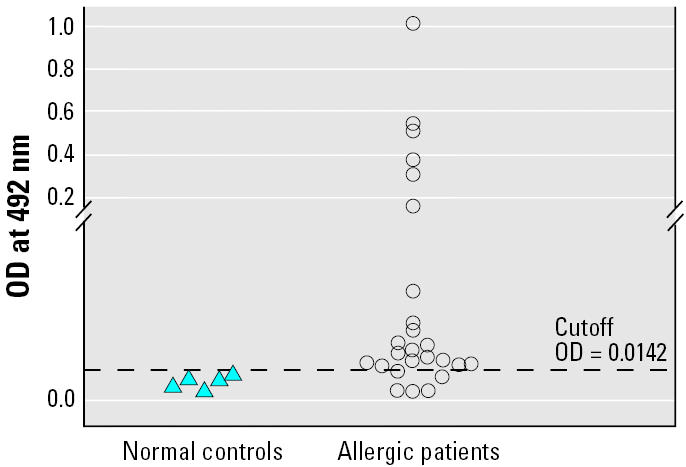
Scatter plots of sandwich ELISA results for detecting specific IgE to *P. americana* arginine kinase in sera of allergic patients and normal controls.

The MAb38G6 and the affinity-column–purified arginine kinase were used in the sandwich ELISA to detect arginine-kinase–specific IgE in serum samples of 25 pediatric patients and five normal controls. It was found that 20 of 25 patients (80%) gave positive sandwich ELISA results; none of the serum samples of nonallergic individuals were positive (Figure 5).

## Discussion

Although CR allergy is a public health problem of Thailand, neither the precise nature nor the source of the CR allergens has been thoroughly studied. In Thailand, the predominant CR species is the American cockroach, *P. americana* (Tungtrongchitr et al. 2004). Available information from elsewhere suggests that the allergens may derive from several CR anatomical parts as well as from excretions and secretions, such as saliva, fecal material, and regurgitated secretions (Menon et al. 1991; Richman et al. 1984). Numerous protein components in the *P. americana* crude extract were found to react to the IgE in sera of CR-allergic Taiwanese patients (Wu et al. 1997). These components ranged from > 120 down to approximately 6 kDa in molecular mass (Wu et al. 1996). Among them, the 78, 72-, 45-, and 28-kDa components were most reactive to the patients’ serum IgE, implying their high allergenicity. CR allergens, such as *P. americana* Per a 1 and variants, tend to exist in multimeric forms (Pomes et al. 2002). Thus, several antigen–antibody reactive bands of different molecular masses are usually seen in Western blot analyses of SDS-PAGE–separated CR allergens and IgE in sera of CR-allergic patients. In 2003, we produced two hybridoma clones, 3C2 and 38G6, which secreted MAb reactive only to antigens in *P. americana* crude extract. The specific antigen of the MAb3C2 proved to be a Per a 1 variant, Per a 1.0105, which is a 124-aminoacid protein with a molecular mass of 13.8 kDa (Diraphat et al. 2003). However, the MAb3C2 showed an antigen–antibody reactive band at approximately 45–40 kDa in the Western blot analysis against SDS-PAGE–separated crude *P. americana* extract (Sookrung et al. 2003), indicating the allergen’s polymeric form. The epitope of the MAb38G6 and the nature and function of the *P. americana* protein carrying the epitope have not yet been studied.

Random peptide phage display libraries have been successfully applied for mapping epitopes of various MAb specific to various pathogens, such as bovine herpes virus (Lehman et al. 2004) and *Neisseria meningitides* (Charalambous and Feavers 2000). A random peptide phage library designated T7S constructed by G. Froman (Department of Microbiology, Uppsala University, Sweden), was used previously for the determination and characterization of a mimotope that bound to MAb to mouse polyomavirus large T-antigen (Houshmand et al. 1999) and a mimotope of MAb specific to *Leptospira* spp. (Tungtrakanpong et al., in press). In this study, the library was used successfully in a search for a mimotope of MAb to *P. americana* allergen, secreted from the hybridoma clone 38G6 [protein purified from American CR extract that bound to this MAb was shown to be an allergen (Sookrung et al. 2003)]. All of the 10 isolated phage clones derived from the fourth phage sublibrary that showed a high binding affinity to the immobilized MAb38G6 revealed the same nucleotide sequence in their genome, and hence the same encoded polypeptide (i.e., LTPCRNK) displayed on their surface, implying the high specificity of this peptide sequence to the MAb38G6. Although the LTP is a part of the T7 capsid protein (Houshmand et al. 1999; Tungtrakanpong et al., in press), it can be also a part of the mimotope. By comparing the mimotope amino acid sequence with those in the database using BLASTp (protein–protein BLAST) software (NCBI 2005 ), it was revealed that the mimotope had an 83–100% identity with various proteins of the insect phylogeny. Although this mimotope search did not give an account on the function of the mimotope-containing protein, nevertheless, the findings implied that the *P. americana* protein that contained the mimotope/epitope to MAb38G6 was a pan-insect protein. This protein was previously shown to be an allergen by a P-K test performed in guinea pigs sensitized with a pool of sera of CR-allergic patients, as described previously. Identification of the MAb38G6-bound protein by LC-MS revealed that the protein is the *P. americana* arginine kinase. The LC-MS result could be confirmed by MS-PMF and BLAST search. Heterologous arginine kinases have previously been demonstrated to be cross-reactive pan-invertebrate allergens (Binder et al. 2001). The native arginine kinase purified from *P. americana* extract reacted to IgE in 80% and 100% of the CR-allergic Thai patients by sandwich ELISA and Western blotting, respectively. Our data demonstrate that the *P. americana* protein arginine kinase is a major CR allergen. Allegenicity of the affinity-purified or recombinant arginine kinase should be evaluated *in vivo* by performing skin prick tests in the CR-allergic patients.

## Figures and Tables

**Table 1:** Characteristics of the 25 CR-allergic patients and the five normal controls.

Patient/control no.	Age (years)	Sex	Specific IgE to German CR extract tested by CAP system (IU)	Skin prick test positive to allergen(s)	Diagnosis
P1	24	M	ND	CR, house dust mite, ant	AR
P2	22	F	ND	CR, mosquito, housefly, pollen	AR
P3	24	M	ND	CR, grass, cat, house dust mite	AR
P4	15	M	ND	CR, grass, house dust mite, cladospore	AR
P5	22	M	ND	CR, house dust mite	AR
P6	27	M	ND	CR	AR
P7	23	M	ND	CR, dog, cat, house dust mite	AR
P8	32	M	2.86	CR	AR
P9	40	M	2.17	CR	AR
P10	28	F	3.95	CR	Atopic dermatitis
P11	27	F	0.42	CR	AR
P12	32	M	ND	CR	AR
P13	26	M	ND	CR	AR
P14	29	F	ND	CR	AR
P15	30	F	ND	CR	AR
P16	46	M	ND	CR	AR
P17	30	F	ND	CR, house dust mite	AR, Asthma
P18	21	F	ND	CR, grass, dog, cat, house dust mite	AR
P19	21	F	ND	CR, grass, dog, cat, house dust mite	AR
P20	21	F	ND	CR	AR
P21	22	F	ND	CR, house dust mite	AR
P22	21	F	ND	CR	AR
P23	22	M	ND	CR, house dust mite	AR
P24	22	F	ND	CR, house dust mite	AR
P25	22	M	ND	CR, house dust mite	AR
C1	20	F	ND	—	Normal
C2	21	M	ND	—	Normal
C3	20	F	ND	—	Normal
C4	20	M	ND	—	Normal
C5	21	F	ND	—	Normal

Abbreviations: —, negative; AR, allergic rhinitis; C, control; CR, both American and German CR extracts; F, female; M, male; ND, not done; P, patient.

**Table 2:** Mimotope of MAb38G6 compared with peptide sequences in the protein database.

Mimotope peptide sequence	Peptide in database	Identity (%)	Ortholog protein
TPCRN	TPCRN	100	ENSANGP00000010401 protein of *Anopheles gambiae*
TPCRN	TPCRN	100	Notch homolog scalloped wings of sheep blow flies (*Lucilia cuprina*)
TPCRN	TPCRN	100	Delta protein of the honey bee (*Apis mellifera*)
PCRNK	PCRNK	100	Neu5Ac synthase of *Drosophila melanogaster*
PCRNK	PCRNK	100	GA18754-PA protein of *Drosophila pseudoobscura*
LTPCRN	LTPCRD	83	Tyrosine phosphatase of *Drosophila melanogaster*
LTPCRN	MTPCRN	83	Delta protein of honey bee (*Apis mellifera*)
